# Retraction: Interferon gamma peptidomimetic targeted to interstitial myofibroblasts attenuates renal fibrosis after unilateral ureteral obstruction in mice

**DOI:** 10.18632/oncotarget.28532

**Published:** 2024-01-24

**Authors:** Fariba Poosti, Ruchi Bansal, Saleh Yazdani, Jai Prakash, Leonie Beljaars, Jacob van den Born, Martin H. de Borst, Harry van Goor, Jan-Luuk Hillebrands, Klaas Poelstra

**Affiliations:** ^1^Department of Pathology and Medical Biology, Division of Pathology, University Medical Center Groningen, University of Groningen, Groningen, The Netherlands; ^2^Department of Biomaterials Science and Technology, Division of Targeted Therapeutics, MIRA Institute, University of Twente, Enschede, The Netherlands; ^3^Department of Pharmacokinetics, Toxicology and Targeting, University of Groningen, Groningen, The Netherlands; ^4^Department of Internal Medicine, Division of Nephrology, University Medical Center Groningen, University of Groningen, Groningen, The Netherlands; ^5^Department of Microbiology and Immunology, Laboratory of Molecular Immunology, Rega Institute, KU Leuven, Belgium; ^*^Share senior authorship

**This article has been retracted:** This article contains duplicated images found in a previously published paper. Specifically, images of a-SMA expression and fibronectin expression in Figures 3B and 4A were duplicated from Figures 5C and 6C in [1]. Therefore, with the agreement of all authors, Oncotarget has decided to retract this paper.

Original article: Oncotarget. 2016; 7:54240–54252. 54240-54252. https://doi.org/10.18632/oncotarget.11095
